# Facet Control of Gold Nanoplate on Sacrificial Transition Metal Dichalcogenides

**DOI:** 10.1002/smtd.202401720

**Published:** 2025-04-25

**Authors:** Ka Ho Leung, Lok Wing Wong, Ping Man, Shan Gao, Shan Jiang, Lingli Huang, Tianren Chen, Jiong Zhao, Thuc Hue Ly

**Affiliations:** ^1^ Department of Chemistry Centre of Super Diamond & Advanced Films (COSDAF) State Key Laboratory of Marine Pollution City University of Hong Kong Kowloon 999077 P. R. China; ^2^ City University of Hong Kong Shenzhen Research Institute Shenzhen 518057 P. R. China; ^3^ Department of Applied Physics The Hong Kong Polytechnic University Kowloon Hong Kong 999077 P. R. China; ^4^ Department of Chemistry, Centre of Super Diamond & Advanced Films (COSDAF) City University of Hong Kong Kowloon 999077 P. R. China; ^5^ The Hong Kong Polytechnic University Shenzhen Research Institute Shenzhen 518057 P. R. China

**Keywords:** epitaxial growth, gold nanostructures, transition metal dichalcogenides

## Abstract

The synthesis of gold nanostructures (AuNS) is a classical topic, celebrated for the exceptional capabilities these structures exhibit across a spectrum of applications. Controlling the morphology and location of gold nanostructures on a large scale is typically a complex task. For example, the thermal annealing method necessitates precise management of the wetting behavior between the gold nanofilm and the substrate during heating to facilitate the transformation of the gold nanofilm into gold nanostructures. Here, the study innovatively applies 2D monolayer MoS_2_ as the sacrificial substrate for gold nanostructure growth, leveraging the epitaxy between MoS_2_ and gold. This relationship leads to the patterning of larger and more uniform gold nanostructures when contrasted with those grown on original SiO_2_/Si substrates. Moreover, the gold nanostructures prepared under this method present four times enhancement in photoluminescence signal of AuNS/TMDC heterostructure, demonstrating the potential application on optoelectronics.

## Graphical abstract

Annealing gold nanofilm is an effortless way to prepare gold nanostructures. In this study, the utilization of sacrificial 2D transition metal dichalcogenides promotes such a process allowing the formation of target gold nanostructures provided in reduced thickness and shows potential in plasmonic application.

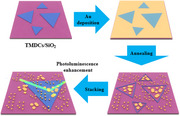

## 1 Introduction

Gold, among the most renowned metals on Earth, is esteemed for its distinctive color and exceptional stability in everyday use. Furthermore, scientific exploration of gold and gold nanostructures is highly regarded for their exceptional performance across a multitude of applications, including medicine,^[^ ^1^, ^2^, ^3^ ^]^ catalyst,^[^ ^4^, ^5^, ^6^ ^]^ environmental,^[^ ^7^, ^8^ ^]^ and plasmonic.^[^ ^9^, ^10^ ^]^ The effectiveness of gold nanostructures in these domains is significantly influenced by several factors, such as shape, size, and crystal structures.^[^ ^11^, ^12^, ^13^, ^14^, ^15^ ^]^ To meet the demand for precise control over gold synthesis, various methodologies have been documented, predominantly revolving around wet chemical techniques such as Turkevich, Brust‐Schiffrin, and seed‐mediated growth.^[^ ^16^, ^17^, ^18^ ^]^

Apart from wet chemical methods, the preparation of gold nanostructures and more importantly, their subsequent patterning process can also be achieved by physical processes. By employing techniques like e‐beam or thermal deposition of gold nanofilm followed by thermal treatment, it can lead to the formation of distinct gold nanostructures from the initial film. The effectiveness of this transformation is significantly influenced by several factors, including the annealing temperature, which can dictate the size and shape of the resulting nanostructures, as well as the thickness of the gold nanofilm, which determines the initial amount of material available for structuring. By carefully controlling these parameters, researchers can achieve a wide range of gold nanostructures that can be tailored for specific applications in areas such as electronics and photonics.^[^ ^19^, ^20^, ^21^, ^22^ ^]^

In addition to the previously mentioned applications, gold plays a crucial role in the synthesis of various nanomaterials, notably in the fabrication of 2D transition metal dichalcogenides (TMDCs). Gold nanostructures frequently act as substrates or seeds that guide and control the synthesis of these 2D materials.

For instance, in the process of creating thin films, gold nanostructures can induce orientation‐directed growth, ensuring that the resulting layers align in a specific manner.^[^ ^23^ ^]^ Similarly, particle‐like structures of gold can direct the growth of nanotubes, allowing for tailored properties and functionalities in the final products.^[^ ^24^ ^]^ Furthermore, precise patterning technique is employed to control the seeding process, leading to enhanced uniformity and scalability in the synthesis of TMDCs.^[^ ^25^ ^]^

The success of these methods is largely attributed to the advantageous properties of gold, particularly its shared crystal structure with certain TMDCs and the robust interaction between sulfur and gold.^[^ ^26^, ^27^ ^]^ This interaction facilitates the epitaxial growth of sulfur‐based TMDCs, such as molybdenum disulfide (MoS₂) and tungsten disulfide (WS₂). The compatibility of gold nanostructures with these materials not only improves the quality of the TMDCs produced but also opens up new avenues for exploring the reciprocal influence, where gold nanostructures are tailored based on the properties of the TMDCs being synthesized.

Hereby we report the use of 2D monolayer MoS_2_ as the sacrificial epitaxial substrate, which can guide and accelerate the crystallization of sputtered gold nanofilm, allowing the patterning of more regular and larger‐sized gold nanostructures compared to non‐crystalline SiO_2_/Si surface. The MoS_2_‐guided gold nanostructures present a significant enhancement in photoluminescence (PL) signal of AuNS/MoS_2_ heterostructure, demonstrating the potential of plasmonic applications.

## 2 Results and Discussion

### 2.1 Epitaxial Growth of Gold Nanostructures and Characterizations

**Figure 1:**
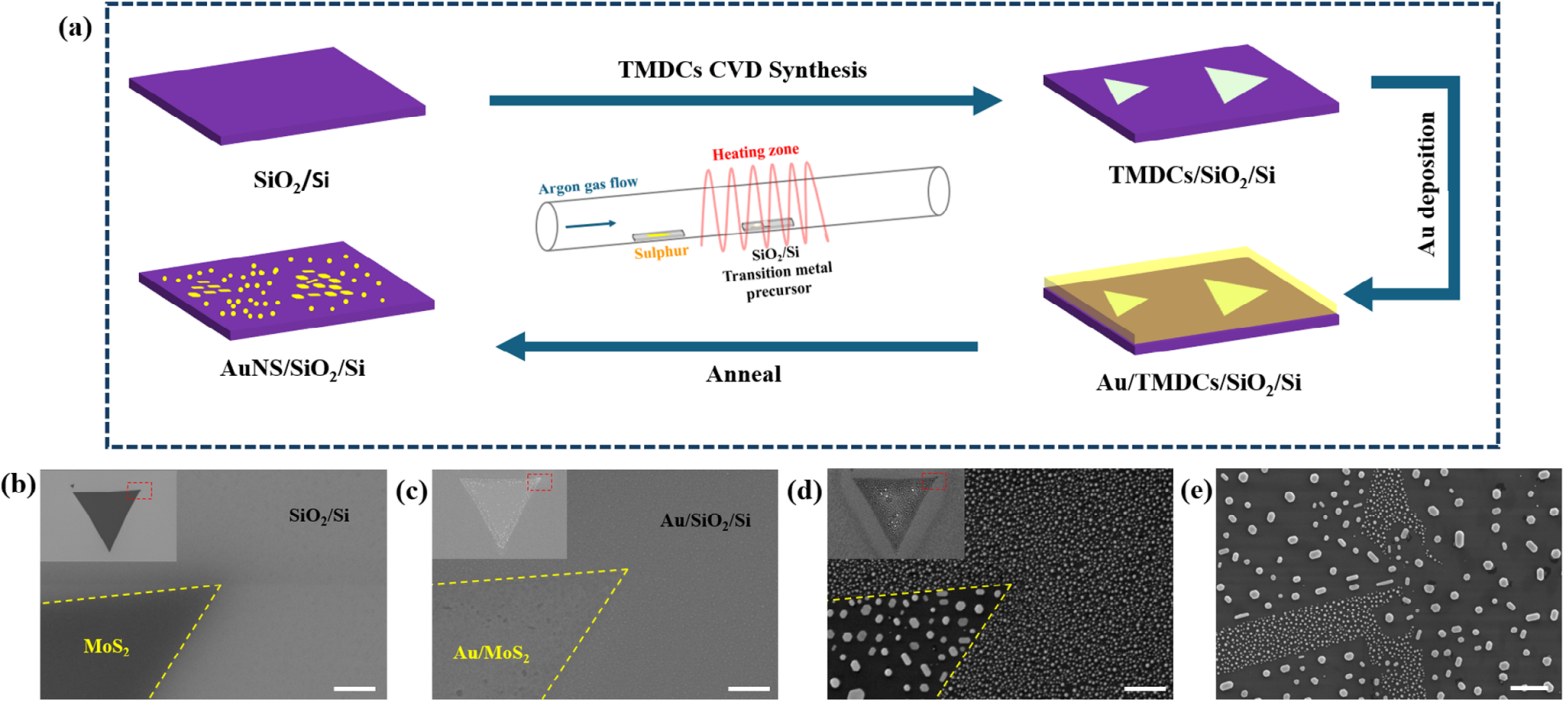
a) Schematic flow for the synthesis of MoS_2_ flakes and preparation of gold nanostructures, SEM image of b) MoS_2_/SiO_2_/Si, c) Au/MoS_2_/SiO_2_/Si, d) AuNS/SiO_2_/Si using in‐house CVD growth MoS_2_, and e) annealed AuNS/SiO_2_/Si prepared using mechanically exfoliated MoS_2_, scale bars, 1 µm.

As illustrated in the methods section and schematic flow in **Figure** **1**a, the gold nanostructures were prepared by epitaxial growth on transition metal dichalcogenides. 2D monolayer MoS_2_ was first synthesized using chemical vapor deposition on a SiO_2_/Si substrate. This was followed by gold sputtering and annealing, which resulted in the formation of various gold nanostructures on the substrate. These structures are categorized based on their size and morphology into two types: those formed on the MoS_2_ layer position (sacrificed‐MoS_2_ AuNS) and those on the SiO_2_/Si substrate surface.

To demonstrate the ease of scale up process, the gold nanofilm is deposited through sputtering method. Limited by the performance of sputtering machine and to minimize error, the deposition time is limited to 10 seconds with the measured thickness is ≈5 nm according to the AFM topography (Figure S1, Supporting Information). Apart from the original nanofilm thickness, we conduct series of annealing with varying temperatures and time to study the formation process of gold nanostructures (Figure S2, Supporting Information).

Starting at 200 °C, the gold film remains intact in both regions, with no significant changes observed. As the temperature increases, the gold in direct contact with SiO_2_/Si begins to melt, forming a hemispheric shape, while the gold in contact with MoS₂ retains its sputtered film structure. At 400 °C, the gold on MoS_2_ develops a unique network‐like morphology, exposing parts of the SiO_2_/Si substrate in between, while the central area remains intact. This trend indicates that the formation of gold nanostructures on the MoS_2_ surface requires higher energy, originating from the edges of MoS_2_ and progressing toward the center. Consequently, 600 °C is selected as the minimum temperature necessary to form rigid gold nanostructures using this method.

At this temperature, a series of time variations is studied to examine the effect of annealing duration. After annealing at 600 °C for 1 and 5 minutes, the gold nanostructures formed on the MoS_2_ surface are less rigid and have more curved edges compared to those subjected to longer annealing times. This suggests that not only temperature but also time significantly affects the morphology of gold nanostructures. Based on these findings, we determined that the synthesis parameters for gold nanostructures in the subsequent part of our work will be an annealing temperature of 600 °C for 20 minutes.

Notably, the melting point of gold nanofilm is significantly reduced compared to the melting point of bulk gold which exceeds 1000 °C. This phenomenon is known as the size‐dependent melting point depression.^[^ ^28^ ^]^ When the size of material is reduced to nanoscale, its surface‐to‐volume ratio increases substantially. The rapid change leads to surface effects, including variations in surface energy and surface tension., In addition to the lattice structure of nanosolids, these factors result in change in melting behavior of material.^[^ ^29^, ^30^ ^]^

SEM images in Figure 1b–d show the in situ evolution of single MoS_2_ flake throughout the process, the clear contrast difference in Figure 1b shows the synthesized MoS_2_ on SiO_2_/Si, which the darker part is the MoS_2_ flake with lower conductivity compared to SiO_2_/Si. After sputtered with gold, the whole substrate surface is covered and thus no significant contrast between MoS_2_ and SiO_2_/Si is observed (Figure 1c). After the annealing process, the gold film turned into nanostructures, with the MoS_2_ region labeled in Figure 1d shows the distribution difference between MoS_2_ and SiO_2_/Si, in which this difference is also observed on exfoliated MoS_2_ transferred to SiO_2_/Si substrate (Figure 1e). Comparing the gold nanostructures formed on MoS_2_ and SiO_2_/Si, it is evident that the sacrificed‐MoS_2_ AuNS exhibit more regular shapes and appear larger in size, primarily appearing rod‐like or hexagonal form. In contrast, the gold nanostructures formed on SiO_2_/Si are generally smaller and spherical in shape. We also measured the height of the gold nanostructures using AFM topography and compared these measurements to the sputtering height (Figure S1, Supporting Information). The synthesized MoS_2_ monolayer was examined by Raman spectroscopy (Figure S3, Supporting Information), apart from the characteristic E^1^_2g_ and A_1g_ peak of MoS_2_, the inter peak distance in Raman spectrum is used to determine the number of layers of MoS_2_, in which the distance is 18.3 cm^−1^, confirming the single‐layered nature of the synthesized MoS_2_.

**Figure 2:**
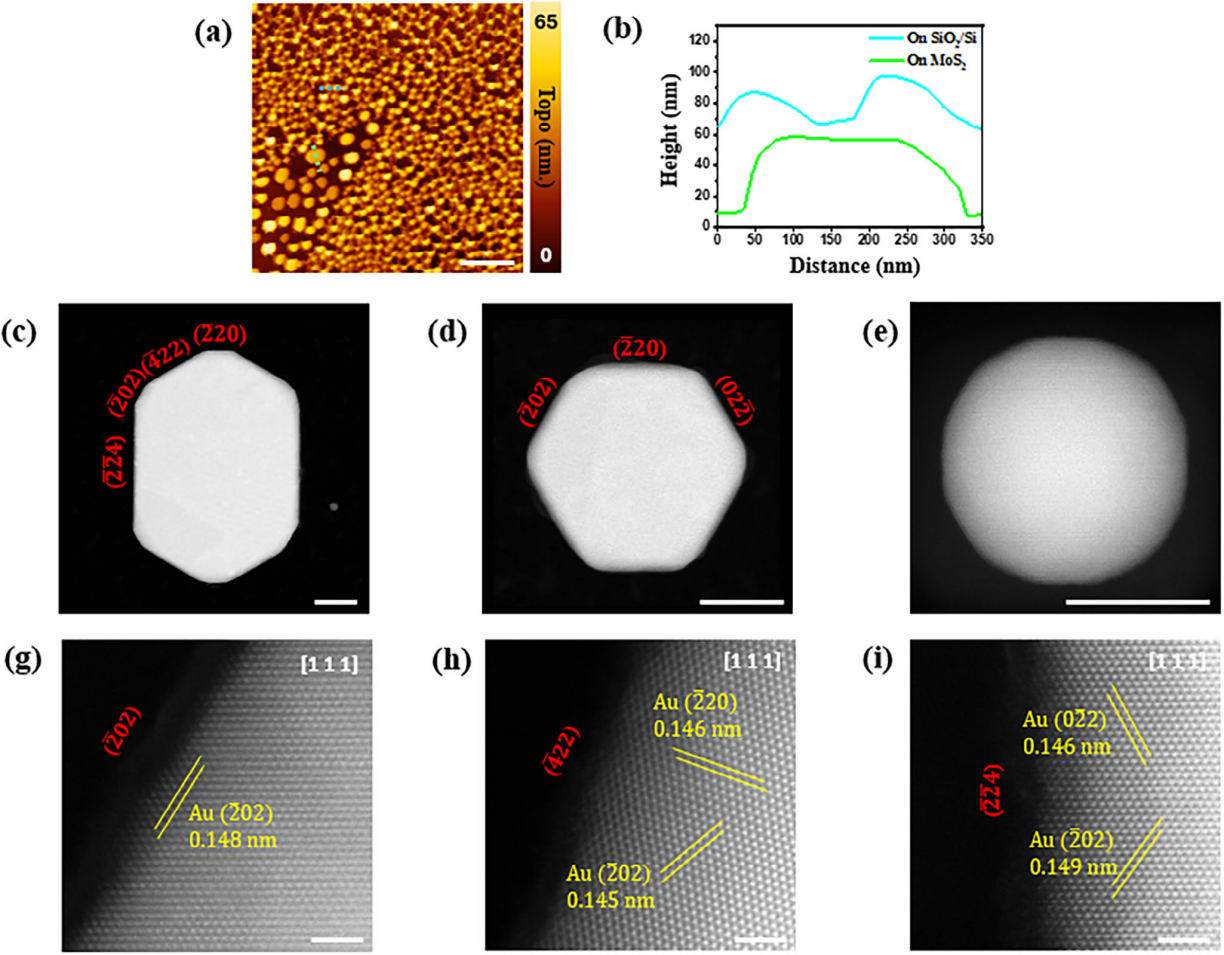
a) AFM topography images of AuNS, scale bar, 1 µm, and b) height profile of gold nanostructures labeled in (a). HAADF‐STEM images of c,d) sacrificed‐MoS_2_ AuNS and e) AuNS formed on SiO_2_/Si, scale bars, 30 nm, and high‐resolution HAADF‐STEM images from (c) to (g) $\left(\bar{2}02\right)$, h) $(\bar{4}22),$ and i) $\left(\bar{22}4\right)$ surfaces, scale bars, 1 nm.

Owing to the nanometer size of gold nanostructures and the limitation of the resolution of optical microscopy, the gold nanostructures were examined by scanning electron microscope and atomic force microscope for more detailed observation. It was found that the height of sacrificed‐MoS_2_ AuNS increased from 5 nm to 40–55 nm (**Figure** **2**a,b), similar to the structures formed on SiO_2_/Si surface. This indicates that the sputtered gold melts and aggerates to form various gold nanostructures during annealing. Previous reports have shown that the deposited thickness of gold is related to the final shape of the gold nanostructures after such heat treatment, with approximately 8–10 nm of original gold nanofilm required to achieve a similar morphology compared to this study.^[^ ^21^, ^31^, ^32^ ^]^ In our work, we demonstrated that the sacrificial 2D TMDCs enable preparation of target gold nanostructures with reduced initial thickness and the distribution of these nanostructures is completely related to the MoS_2_ presence on the same substrate.

To elucidate the correlation between the formation of gold nanostructures and TMDCs, additional TMDCs were employed for comparative analysis. Specifically, 2D WS_2_ and ReS_2_ were utilized for gold nanostructure synthesis following this proposed scheme, and the resultant structures were examined under SEM (Figure S4, Supporting Information). It is evident that similar results were achieved only with WS_2_, not with ReS_2_. Although MoS_2_, WS_2_, and ReS_2_ belong to the same category of sulfide‐based TMDCs, the differences in results can be attributed to their distinct crystal structures. Both MoS_2_ and WS_2_, as 2H‐phase crystals, exhibit similar structures with minor variations in lattice constants due to the slight atomic size difference between Mo and W. In contrast, ReS_2_, in its 1T' phase, possesses a distinctly different atomic arrangement. The observed divergence in outcomes is explained by the 2H‐phase materials acting as nucleation sites, which expedite the crystallization of gold nanostructures due to the inherent characteristics of the 2H‐phase lattice. Previous researches have explored the epitaxial relationship between face‐centered cubic (fcc) Au and 2H‐MoS_2_, more specifically, the [111] plane of Au and [001] plane of MoS_2_.^[^ ^33^, ^34^ ^]^ Reported by Cui et al., the lattice mismatch between Au [220] planes and MoS_2_ [110] planes yields a Moire pattern with spacing |Δ**g**_a_|^−1^ equal to 1.63 nm and observed under TEM.^[^ ^35^ ^]^ The epitaxial relationship between MoS_2_ and Au explains the MoS_2_ layer underneath gold nanofilm with small lattice mismatch serves as a template substrate that guides the arrangement of gold atoms and thus promotes the formation of hexagonal AuNS.

This preparation method is not limited to TMDCs grown by CVD; MoS_2_ produced via mechanical exfoliation also demonstrates similar results, as shown in Figure 1e, thus eliminating the substrate effects associated with CVD growth. In addition to the possible cause of wetting behavior difference between MoS_2_ and SiO_2_/Si, a set of gold nanofilm thickness experiment was conducted. Various thickness of gold nanofilm were prepared through sputtering and annealed under 600 °C as illustrated in Methods. Unlike significant difference in terms of the morphology of AuNS between MoS_2_ and SiO_2_/Si, similar hexagonal AuNS can also be found on SiO_2_/Si substrate when gold nanofilm thickness increases (Figure S5e,f, Supporting Information). Thus, we further increase the thickness of sputtered gold nanofilm to ≈27 nm, as turns out not only the AuNS on SiO_2_/Si, but also some of the AuNS in previous MoS_2_ region are irregular (Figure S5h,i, Supporting Information), nevertheless, hexagonal AuNS can be located on both region. The result demonstrates control of AuNS morphology can be achieved by controlling gold nanofilm thickness on SiO_2_/Si and as reported on other substrates, proves the formation of rigid AuNS is not originated from the wetting behavior difference between MoS_2_ and SiO_2_/Si.

The AuNS prepared with different initial gold nanofilm thickness is further studied using AFM as shown in Figure S6 (Supporting Information). Compared to Figure 2a, as gold nanofilm initial thickness increases, the thickness and diameter of AuNS are found to increase simultaneously, and the increase is found to be more significant for AuNS formed on SiO_2_/Si. The increase in dimension of AuNS will reduce the plasmonic performance due to increased travel distance causing more electron energy loss which will be discussed in later part. Nevertheless, this result demonstrates our approach is not only capable to prepare hexagonal AuNS with reduced gold nanofilm thickness compared to novel approach, but also allows preparation of AuNS with smaller dimension affecting the plasmonic performance.

Apart from the morphological differences in gold nanostructures observed under SEM and AFM, we employed transmission electron microscopy (TEM) to examine and compare the detailed crystal structures. As shown in Figure 2c–e, the HDDAF‐STEM image of selected hexagonal‐like, rod‐like, and spherical‐like gold nanostructures are acquired. The selected region in HDDAF‐STEM images labeled in red square was processed through fast Fourier Transformation (FFT) to obtain the FFT pattern (Figure S7, Supporting Information). For both the hexagonal‐like and rod‐like sacrificed‐MoS₂ AuNS, the FFT pattern appears in a hexagonal shape along the [111] axis. By correlating the distance between reflection spots, it is affirmed that both gold nanostructures exhibit a face‐centered cubic (FCC) structure, recognized as the thermodynamically stable form of gold, this result is also confirmed by selected area electron diffraction (SAED) in Figure S7g,h (Supporting Information), the diffraction pattern demonstrates typical FCC structure along [111] axis and proved both AuNS are single crystal. Owing to the small dimension of spherical AuNS, SAED is not applicable to examine single nanostructure. In contrast, the FFT pattern of the nanostructure formed on SiO_2_/Si also aligns with the FCC structure when reflected along the [112] axis. Further structural analysis on sacrificed‐MoS_2_ AuNS are revealed in HAADF‐STEM images as shown in Figure 2g–i, we selected different surfaces of the AuNS to further observe the atomic structure. The high‐resolution STEM images expose the presence of sharp edges with different types of atomic arrangement, including higher index surfaces and these image results agree with the SAED pattern as shown in Figure S7g,h (Supporting Information). Consequently, we have verified that the crystal structures of the gold nanostructures formed on both MoS_2_ and SiO_2_/Si are identical, conforming to the thermodynamically stable form and morphology difference, especially the presence of sharp edges and corners in sacrificed‐MoS_2_ AuNS.

In addition, trace of MoS_2_ is observed under HAADF‐STEM image shown in Figure S8 (Supporting Information). In small portion of sacrificed‐MoS_2_ AuNS, layered structure material can be located on some edges of the AuNS, such layered structure implicates the trace of MoS_2_. Noted that such trace of MoS_2_ was only found in low portion of AuNS and no characteristic signal is found in the Raman spectra, indicating the trace of MoS_2_ could be the remaining MoS_2_ layer while the majority has decomposed during high temperature annealing.

### 2.2 TMDCs/AuNS Photoluminescence (PL) Enhancement Performance

**Figure 3:**
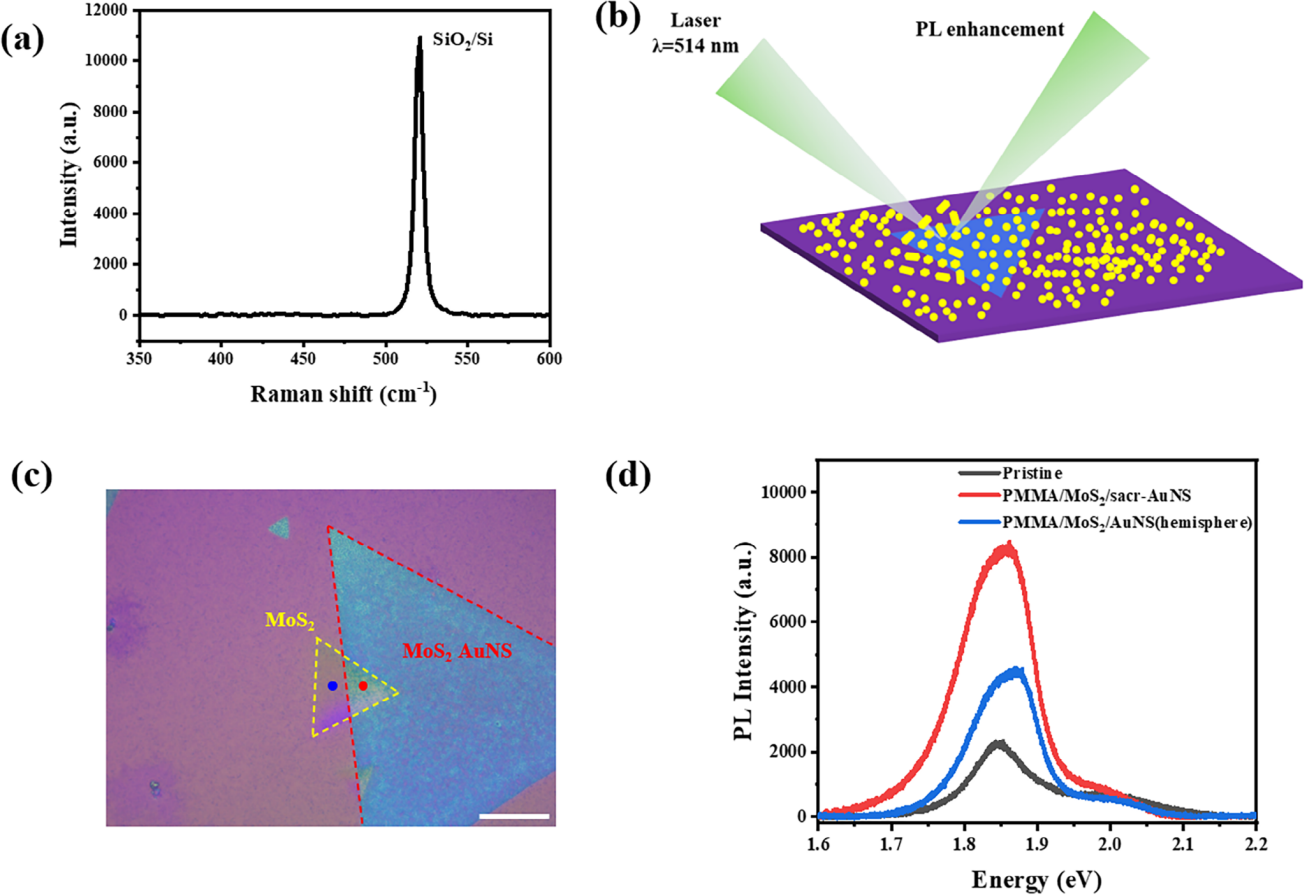
a) Raman spectra taken from hexagonal/rod‐like AuNS, missing of E^1^_2g_ and A_1g_ peaks indicate degradation of MoS_2_. b) Schematic illustration of the photoluminescence (PL) measurement setup. MoS_2_ is transferred through dry transfer method on sacrificed‐MoS_2_ AuNS to examine the performance. c) Optical image and d) single point PL spectrum for pristine MoS_2_ and AuNS/MoS_2_ stacked structure, it is found that stacked with gold nanostructures, PL intensity of MoS_2_ increases, more significantly when stacked with sacrified‐MoS_2_ AuNS, scale bar, 10 µm.

During the annealing process, the synthesized 2D TMDCs degraded, leaving only gold nanostructures on the substrate (**Figure** **3**a). To assess and compare the photoluminescence (PL) enhancement performance of MoS_2_ and the gold nanostructures, MoS_2_ was synthesized on a separate SiO_2_/Si substrate and then stacked onto the AuNS using a dry transfer method to form the MoS_2_/AuNS heterostructure, as shown in the schematic in Figure 3b. The monolayer MoS_2_ flake at the top covered various gold nanostructures, as illustrated in Figure 3c. Part of the flake covered sacrificed‐MoS_2_ AuNS distributed across a triangular shape, while the remaining area covered the AuNS formed on SiO_2_/Si. Dark field optical microscopy (Figure S9a, Supporting Information) provided improved observation of the gold nanostructure distribution and enhanced contrast between the two different AuNS.

The stacked structure was then examined by Raman and photoluminescence spectroscopy. A line scan mapping is employed to measure Raman and PL signal under the excitation of 514 nm laser, as shown in Figure S9 (Supporting Information). The single point PL spectra of MoS_2_ covering different AuNS are selected (Figure 3c) and plotted in Figure 3d. It was found that the PL intensity of the main peak is significantly higher when MoS_2_ covers sacrificial‐MoS₂ AuNS compared to the hemisphere AuNS formed on SiO_2_/Si, with an enhancement of approximately two times. Additionally, the pristine MoS₂ PL spectra before transfer are also plotted. Notably, after being transferred as part of the stacked structure, the PL intensity of MoS_2_ is enhanced regardless of which AuNS is covered, compared to the pristine signal. This demonstrates that both types of AuNS are capable of enhancing the PL signal, albeit to different extents. Furthermore, it is observed that the main peak of the transferred MoS_2_ is slightly blue‐shifted in both regions compared to the pristine sample. Typically, the transfer of MoS_2_ onto such nanostructures induces strain, which is expected to cause a red shift due to changes in the electronic structure.^[^ ^36^ ^]^ However, when the MoS_2_ is transferred to large aggregate, the p‐doping effect dominates the process, leading to a blueshift in the PL spectra, consistent with previous report.^[^ ^37^ ^]^

To ensure the uniformity of transferred MoS_2_ flake, Raman line mapping was analyzed in terms of peak distance and peak ratio, leveraging the unique layer‐dependent Raman characteristics of MoS_2_.^[^ ^38^, ^39^ ^]^ The intrinsic properties of peak distance between E^1^_2g_ and A_1g_ modes in MoS_2_ allow for the determination of the number of layers. The extracted peak distance is similar across the scan region, ranging from 19 to 20 cm^−1^, indicating that the transferred MoS_2_ is monolayer (Figure S9b, Supporting Information). Moreover, the intensity ratio between E^1^_2g_ and A_1g_ is related to the crystallinity of MoS_2_.^[^ ^40^ ^]^ A stable E^1^_2g_/A_1g_ ratio of approximately 0.55 to 0.65 (Figure S9b, Supporting Information) across the scan region indicates no significant difference in crystallinity or defects within the examined MoS_2_ flake. Thus, we can rule out effects from crystallinity and sample quality, concluding that the PL enhancement is attributed to the gold nanostructures.

The enhancement of the PL signal is attributed to the plasmonic interaction between the incident light and the gold nanoparticles, which act as localized plasmonic hot spots. As reported by Wen et al., similar heterostructure of Au/WS_2_ has been studied.^[^ ^41^ ^]^ Under LSPR excitation conditions, additional electrons and excitons are generated by plasmon‐induced charge transfer and field enhancement,^[^ ^42^ ^]^ explaining the PL enhancement in both hemispheres and sacr‐AuNS. Owing to different excitation mechanism, the enhancing ability can be determined by various factors, including the size and shape of AuNS.

Under normal circumstances, the smaller size of the nanoparticles will significantly influence this interaction, leading to a size‐dependent resonance. Although larger particles are able to absorb and scatter more light increasing near‐field intensities, the surface plasmons will tend to decay through photon emission other than exciting electron–hole pairs between AuNS and MoS_2_, originated from the energy loss of electron travelling on metal surface to interface due to electron‐electron scattering.^[^ ^37^, ^43^, ^44^ ^]^ Thus, we should expect higher enhancement of hemisphere AuNS but yet a lower enhancement is observed in the MoS_2_ region integrated with hemisphere AuNS that prepared on SiO_2_/Si surface.

Beyond size, the shape of nanoparticles plays a crucial role in PL enhancement. Several reports have demonstrated that, at the same dimensions, nanoparticles with more rigid shapes and sharper edges yield higher intensity.^[^ ^45^, ^46^, ^47^, ^48^ ^]^ As discussed in previous part relating to AuNS size, our prepared scar‐AuNS is larger sized compared to hemisphere AuNS allowing higher absorption and scatter of light results in increased near‐field intensities. Moreover, the electric field enhancement is maximized at sharp surfaces, making the corners of hexagonal or rod‐like nanoparticles serve as localized hotspots. These localized hotspots contain higher concentration of hot carriers and thus result in greater PL enhancement for gold nanostructures prepared on TMDCs compared to hemisphere gold nanostructures prepared on SiO₂/Si as resulted in the PL spectra in Figure 3d.

## 3 Conclusion

In conclusion, we have demonstrated that the epitaxial growth of gold nanostructures on MoS_2_ accelerates crystallization and restructuring, resulting in more uniform rod‐like and hexagonal nanostructures while enhancing the photoluminescence response of MoS₂. With a well‐defined crystalline structure, the self‐aggregation of gold is more effectively controlled during heat treatment. This study underscores the potential to manipulate morphological properties by leveraging the analogous lattice structures between MoS_2_ and gold nanostructures, thereby enabling further customization of diverse gold nanostructures through this epitaxial growth paradigm.

## 4 Experimental Section

### Preparation of MoS_2_, WS_2_, and ReS_2_

The MoS_2_ flake were synthesized using a single heating zone furnace under atmospheric pressure on 300 nm SiO_2_/Si substrate. Sulfur powder (50 mg) and MoO_3_ (5 mg) serve as the sulfur and molybdenum source respectively. The MoS_2_ was synthesized at 820 °C with the ramping rate of 30 °C min^−1^ under 200 sccm Ar for 15 mins, the furnace then naturally cooled down to room temperature. For synthesis of WS_2_ and ReS_2_, the synthesis condition is similar, with the change of reactant from MoO_3_ to WO_3_ (5 mg) and NH_4_ReO_4_ (1 mg), and the synthesis temperature are set as 840 and 800 °C respectively. Bulk MoS_2_ crystal is purchased from hq graphene and exfoliated using scotch tape method.

### Preparation of Gold Nanostructures on MoS_2_

The SiO_2_/Si substrate with synthesized MoS_2_ flakes were sputtered by nanometers of gold film using an ion sputter(BAL‐TEC, SCD 050), with a working current of 60 mA and sputter time of 10s. The sputtered substrate was then annealed under 600 °C with the ramping rate of 30 °C min^−1^ for 10 mins and cooled down to room temperature naturally under Argon environment.

### Transfer of Gold Nanostructures and MoS_2_

The gold nanostructures/MoS_2_ were transferred by traditional PMMA‐assisted wet transfer. PMMA (10 µm, A4) was spin‐coated on the as‐grown MoS_2_/SiO_2_/Si by 3000 rpm for 60 s. The substrate was then placed floating on the surface of NaOH solution and waited for the PMMA film detached from SiO_2_/Si substrate. The detached PMMA film was placed in deionized water to wash off the remaining ions and finally transferred to TEM grid, and the PMMA was removed by rinsing acetone, isopropanol, and ethanol respectively.

### Fabrication of MoS_2_/Gold Nanostructure Stacked Heterostructure

The stacked heterostructure was prepared using a transfer platform equipped with an optical microscope. SiO_2_/Si substrate with annealed gold nanostructure on SiO_2_/Si substrate was fixed on the transfer platform while the PDMS/PMMA film with MoS_2_ was fixed on the upper half of the transfer platform. With the aid of optical microscope, the position of targeted MoS_2_ and gold nanostructures was located and adjusted to designated position which was controlled separately, the height difference between SiO_2_/Si and PDMS was then reduced subsequently with the trace of the position of MoS_2_ and gold nanostructures until PDMS film firmly attached to the SiO_2_/Si substrate. The platform was heated to 120 °C for 2 min to weaken the adhesive strength of PDMS, and the PDMS layer was slowly removed vertically leaving PMMA film on top of the SiO_2_/Si substrate, as the final structure of PMMA/MoS_2_/gold nanostructures/SiO_2_/Si was prepared.

### Material Characterizations

The MoS_2_ flakes and gold nanostructures were observed through an optical microscope (ZEISS Imager.A2M) under bright field and dark field. Detailed morphology of gold nanostructures was acquired by scanning electron microscope (ThermoFisher, Quattro S), with the spot size of 3.0 and electron beam energy of 10 kV. The topology of gold nanostructures was characterized by atomic force microscopy (Hitachi 5300E) under dynamic force mode with the SI‐DF3 silicon tip. The crystal structure of gold nanostructures was acquired by high‐resolution STEM using a JEM‐ARM200F TEM with a CEOS spherical (Cs) aberration (probe) corrector. The aberration‐corrected STEM image was taken under 300 kV of the accelerating voltage of electron beam. The structural and optical properties were examined by Raman system (Renishaw, inVia confocal), equipped with an excitation laser of 514 nm and grating of 1800 g nm^−1^ under normal operating mode, the measuring parameters were set with an exposure time of 5 s and laser power of 5% respectively unless specified. The photoluminescence spectra of MoS_2_ and stacked structure were measured by the same Raman system with an excitation laser of 514 nm, exposure time for each spectrum was 10s and laser power of 1% respectively unless specified.

## Conflict of Interest

The authors declare no conflict of interest.

## Supporting information



Supporting Information

## Data Availability

The data that support the findings of this study are available from the corresponding author upon reasonable request.
