# Supplementary material for: Facet Control of Gold Nanoplate on Sacrificial Transition Metal Dichalcogenides
Source: Small Methods. 2025 Apr 25;9(8):2401720. doi: 10.1002/smtd.202401720 (PMC12391649; doi:10.1002/smtd.202401720)
Supplement: Supplementary file 1 — Supporting Information [file SMTD-9-2401720-s001.docx]

Supporting Information

Title

Facet control of gold nanoplate on sacrificial transition metal dichalcogenides

Ka Ho Leung, Lok Wing Wong, Ping Man, Shan Gao, Shan Jiang, Lingli Huang, Tianren Chen, Jiong Zhao*, Thuc Hue Ly*


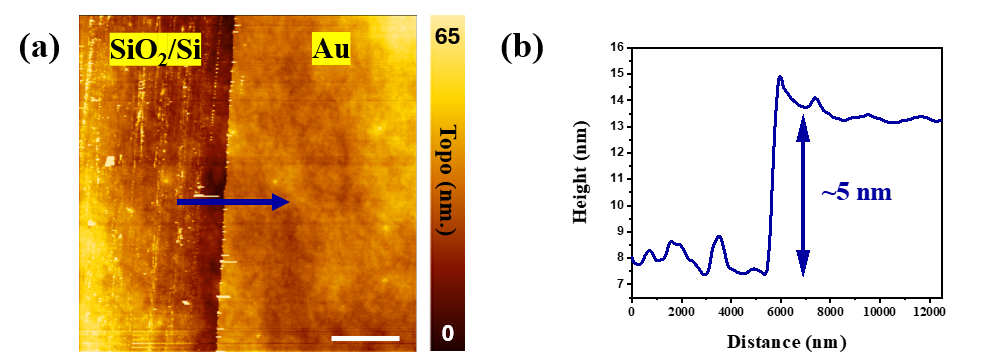


**Figure S1.** (a) AFM Topography image of sputtered Au/SiO_2_/Si, scale bar, 10 µm, and (b) height profile, readouts taken from average height labeled in (a).


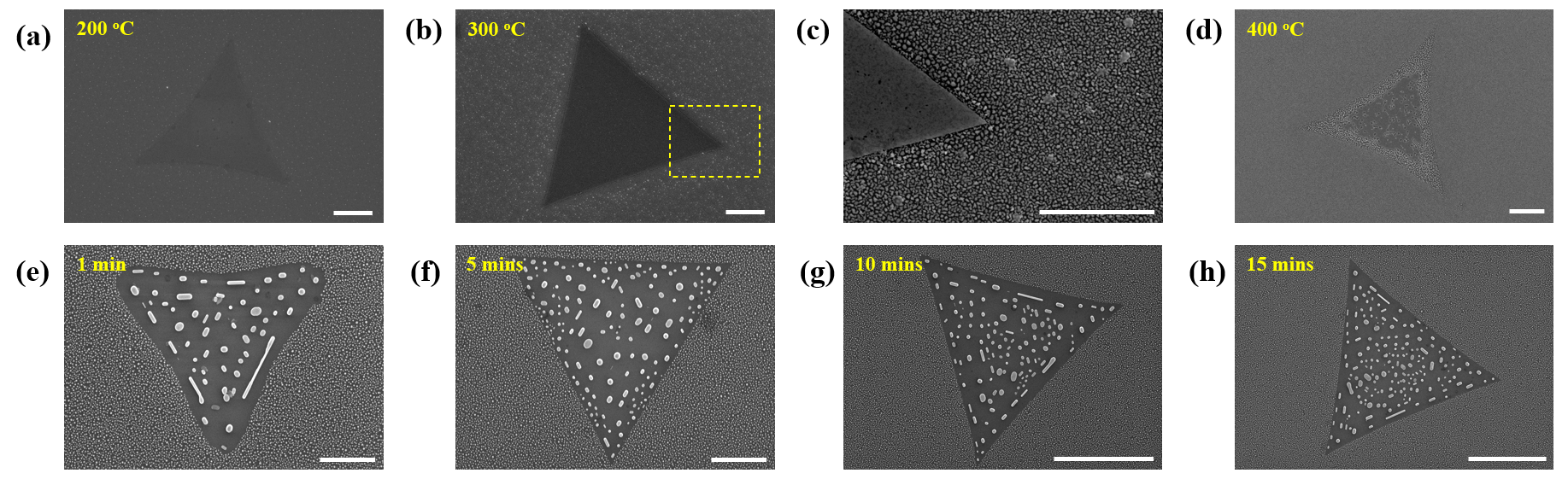


**Figure S2.** (a-d) SEM images on studying the temperature dependance for AuNS formation varying from 200 ^o^C – 400 ^o^C with the heating time set as 20 minutes. With elevating temperature, the gold nanofilm melts at 300 ^o^C forming hemisphere gold nanostructures on SiO_2_/Si, and the image for 400 ^o^C demonstrates the formation of gold nanostructures originating from edge towards center part. (e-h) SEM images on studying the time dependance for AuNS formation under 600 ^o^C varying from 1 min – 20 mins, less rigid and more curvy gold nanostructures are prepared under shorter annealing time, by extending annealing time (>10 minutes), the gold nanostructures appear more rigid in hexagonal/rod-like. Scale bars for (a, b, d, g & h), 10 µm, scale bars for (c, e & f), 3 µm.


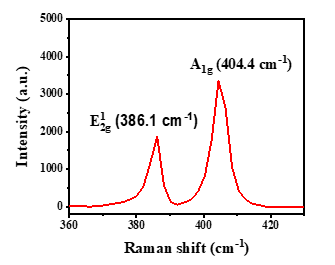


**Figure S3.** (a) Raman spectrum of pristine MoS_2_.


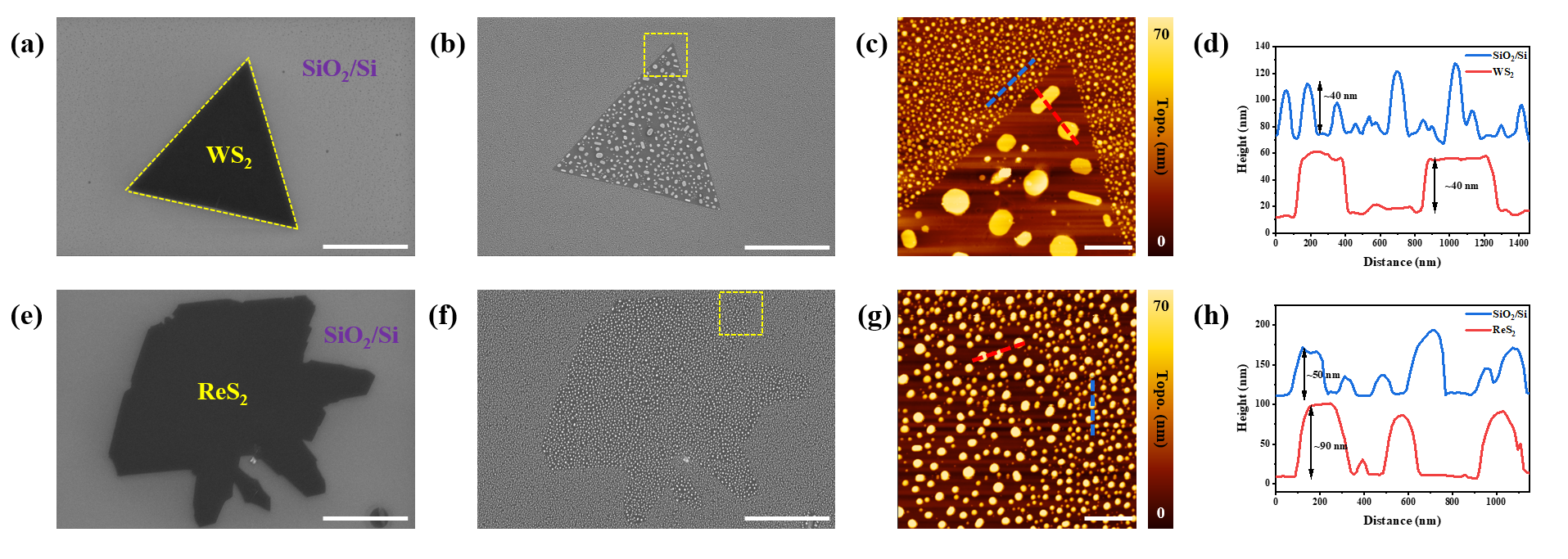


**Figure S4.** SEM images of (a) pristine WS_2_, (b) prepared AuNS, (c) topography image and corresponding (d) height profile. SEM images of (e) pristine ReS_2_, (f) prepared AuNS, (g) topography image and (h) corresponding height profile. Scale bars for (a, b, e & f), 10 µm, scale bars for (c & g), 1 µm.


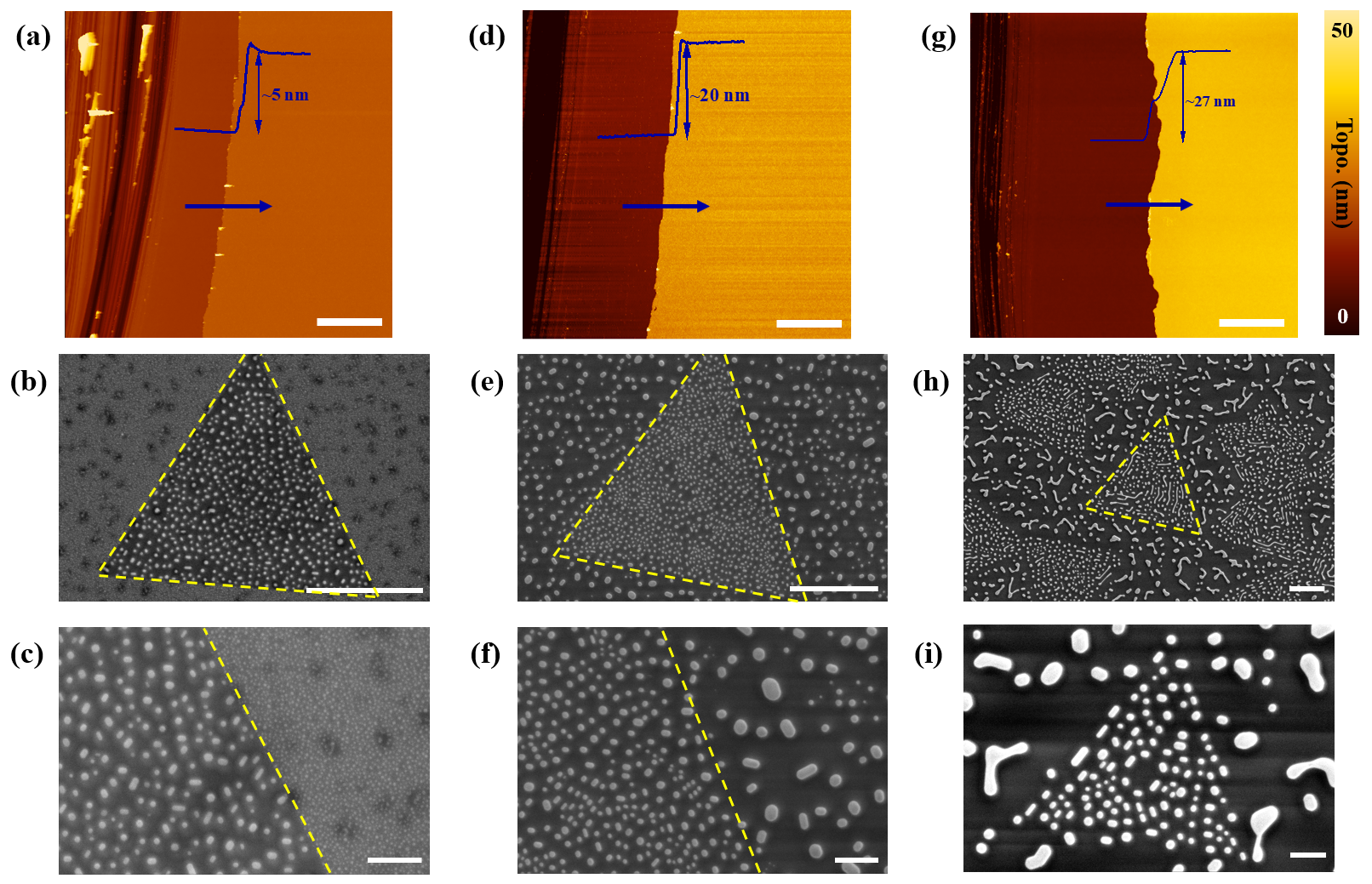


**Figure S5.** Topography image of gold sputtered substrate and SEM images of annealed AuNS with the initial gold nanofilm thickness of (a-c) 5 nm, (d-f) 20 nm and (g-i) 27 nm. Scale bars for (a, b, d, e, g & h), 10 µm, and scale bars for (c, f & i), 2 µm.


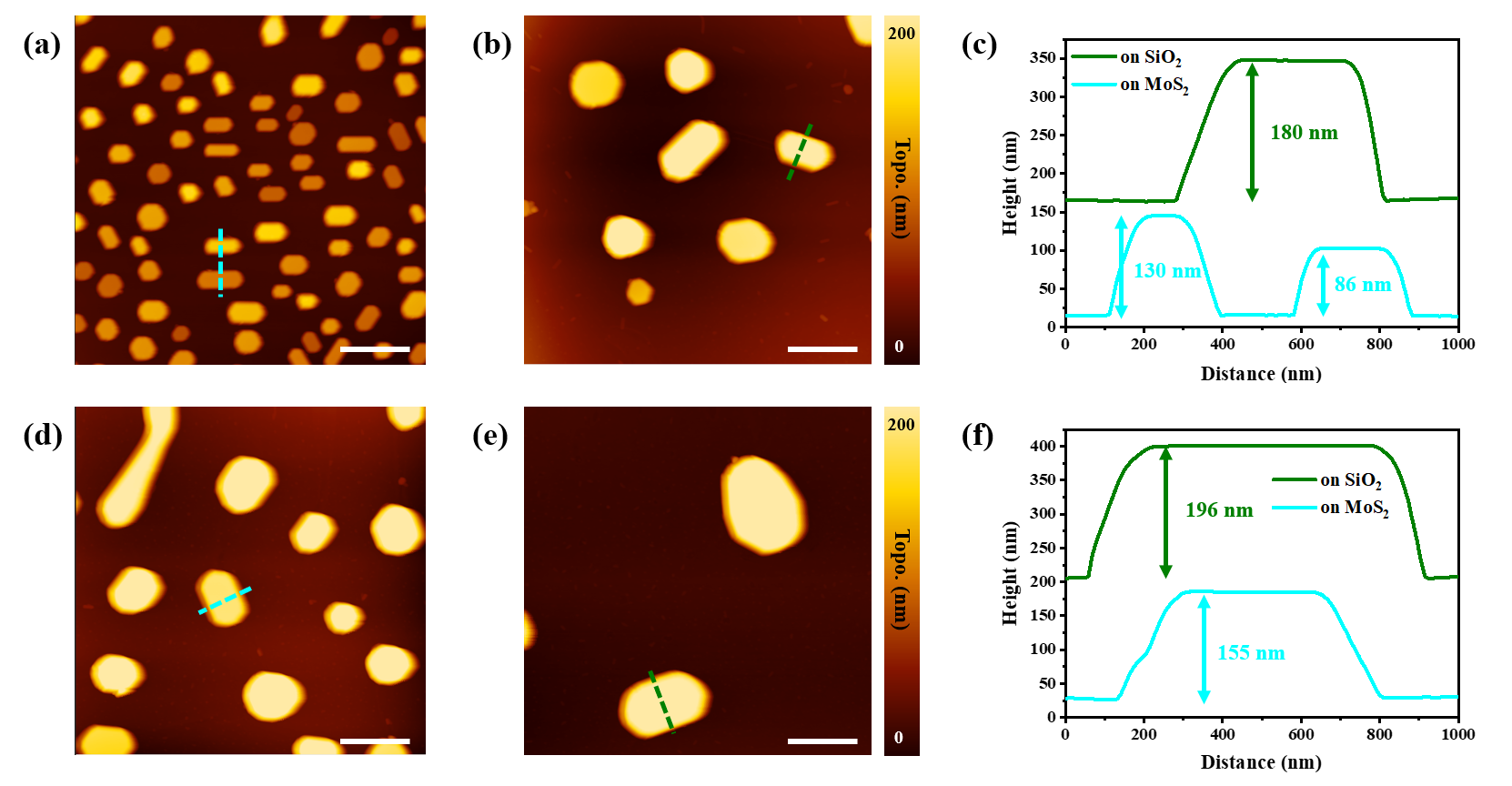


**Figure S6.** Topography image of sacr-AuNS, AuNS formed on SiO_2_/Si and its height profile with initial gold nanofilm thickness of (a-c) 20 nm and (c-f) 27 nm. Scale bars, 1 µm. Noted with the initial gold nanofilm thickness increases, the thickness and diameter of AuNS increases simultaneously.


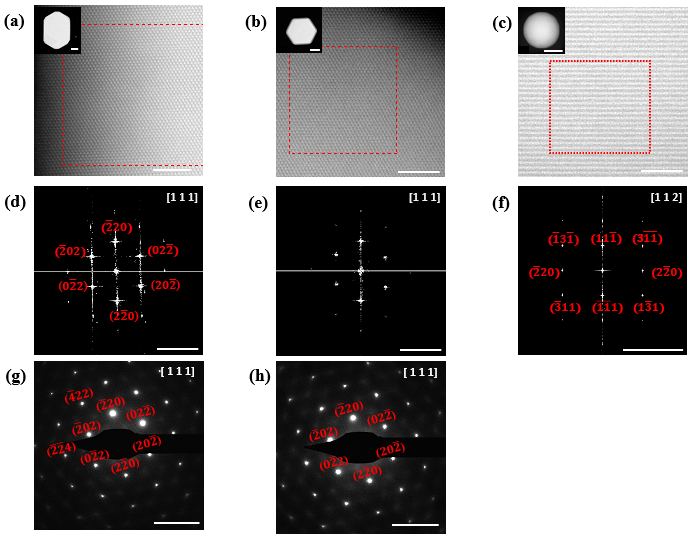


**Figure S7.** HAADF-STEM images of (a, b) sacrififed-MoS_2_ AuNS and (c) AuNS formed on SiO_2_/Si, inset scale bars, 30 nm, and HAADF-STEM image scale bars, 2 nm, (d-f) the corresponding FFT pattern, showing all type of AuNS are FCC structured and (g, h) selected area diffraction pattern (SAED), demonstrates the AuNS are single crystal, scale bars for panel (d-h), 10 nm^-1^.


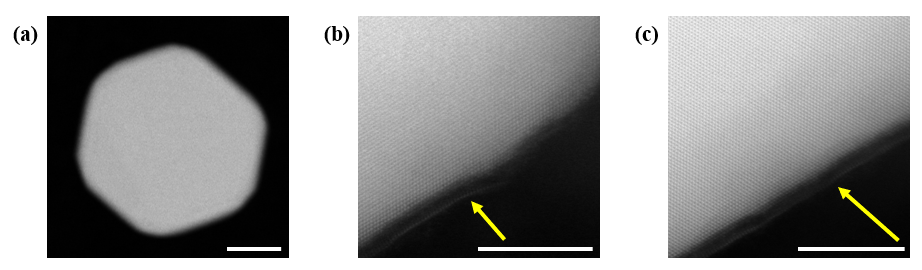


**Figure S8.** (a-c) HAADF-STEM images of sacrififed-MoS_2_ AuNS, noted the yellow arrow denoted the layered structure on the edge of AuNS, shows trace of MoS_2_ layer around the AuNS edge, scale bar for (a), 50 nm, scale bars for (b, c), 5 nm.


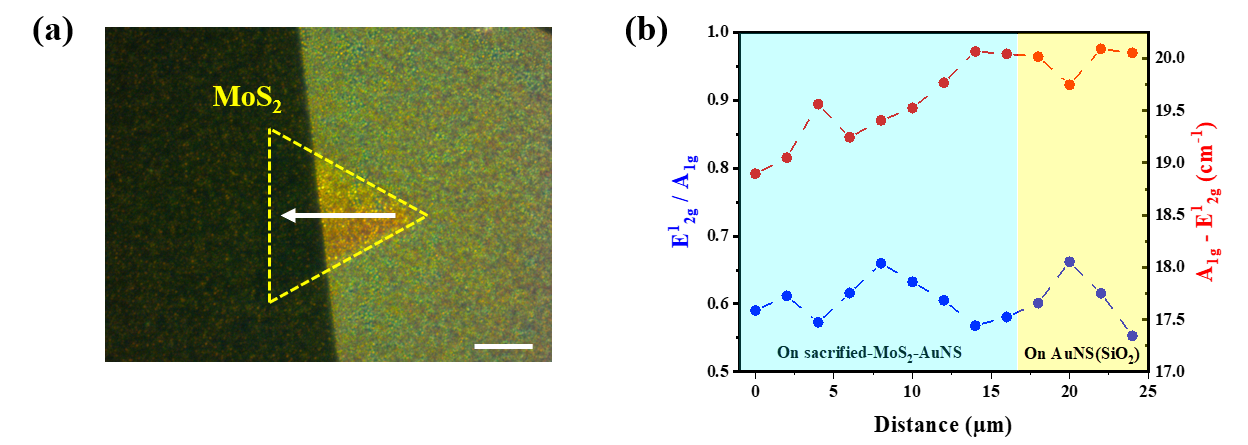


**Figure S9.** (a) Dark field optical image of MoS_2_/AuNS stacked structure, noted the line mapping scan direction labelled, scale bar, 10 µm. (b) E^1^_2g_ and A_1g_ peak distance and peak ratio line mapping, the peak distance is ~19 – 20 cm^-1^, validates that the MoS_2_ is monolayer and the steady peak ratio between E^1^_2g_ and A_1g_ exhibits uniform quality of the MoS_2_ flake which will not affect the PL result.
